# Student-led implementation of Mental Health First Aid training in undergraduate medical education

**DOI:** 10.3389/fpsyt.2026.1893636

**Published:** 2026-09-10

**Authors:** Zoe Mccormack, Khawla Yousif, Elizaveta Abramova, Sourav Arora, Johal Prabnoor, Dolores Keating, Judith Strawbridge

**Affiliations:** 1 School of Pharmacy and Biomolecular Sciences, RCSI University of Medicine and Health Sciences, School of Pharmacy, Dublin, Ireland; 2 School of Medicine, RCSI University of Medicine and Health Sciences, School of Medicine, Dublin, Ireland; 3 Department of Pharmacy, St John of God’s University Hospital, Dublin, Ireland

**Keywords:** curriculum design & development, medical education, mental health, Mental Health First Aid (MHFA), student partnership

## Abstract

**Background:**

Mental health literacy (MHL) is increasingly recognised as a core competency for medical students, yet medical curricula often lack structured teaching on how to recognise and respond to mental health challenges.

**Aim:**

This study was a student-led exploratory study of medical students’ views on the content and delivery of MHFA training, and gathered their suggestions for how MHFA might fit into medical education.

**Methods:**

Using a student partnership design MHFA training was delivered to medical students at the RCSI University of Medicine and Health Sciences (RCSI) across three cohorts between 2023 and 2025. Three post-training focus groups were conducted with 23 participants. Data were analysed using thematic analysis.

**Results:**

Students were highly receptive to the value of MHFA training and viewed mental health skills as essential to their future clinical practice. Three key themes emerged: (1) Mental health literacy as a core competency - students perceived MHFA as filling a crucial curricular gap; (2) MHFA provides a pedagogy of safety and authenticity- the interactive, supportive learning environment encouraged open discussion and reflection; and (3) Optimising MHFA for medical students - participants suggested inclusion of topics such as eating disorders needed to be addressed within mental health education.

**Discussion:**

MHFA training was perceived as relevant, engaging, and valuable by medical students. Participants described MHFA as supporting their confidence in discussing mental health and increasing awareness of wellbeing-related issues. Findings suggest that embedding MHFA within medical education allow students to consider their own mental health literacy, discuss mental health topics with peers, and inquire into their own self-care practices.

## 1 Introduction

Enhancing the capacity of the medical profession to respond to the growing mental health needs of patients is a World Health Organisation (WHO) priority (1). In Ireland, Sharing the Vision emphasises the responsibility of health professionals beyond psychiatry to respond to this need (2). Ensuring that medical graduates have a foundation level of mental health literacy is therefore essential. Mental health literacy refers to knowledge about mental health disorders that supports their recognition, management, and prevention (3).

Concerns regarding medical students’ own mental health further reinforce this need. Medical students are at increased risk of depression, anxiety, and suicide compared with the general population (4–8), despite entering medical training with comparable psychological wellbeing to their peers (9). A Lancet review has called on medical schools to normalise help-seeking and explicitly teach coping skills (10).

Stigma remains an additional barrier. Negative attitudes towards mental illness persist among medical students (11), and while undergraduate education is considered a mechanism for reducing stigma, exposure to psychiatry alone appears insufficient (12).

Mental Health First Aid (MHFA) is one potential educational intervention. MHFA is an internationally recognised programme that equips participants to provide initial support to individuals developing a mental health problem or experiencing a crisis (13, 14). Although originally designed for the general public, MHFA has demonstrated benefits for healthcare students, including nursing, pharmacy, and medicine (15–30). The programme has been shown to improve mental health literacy, confidence, and attitudes while reducing stigma (13, 31–33).

Healthcare professionals often report difficulty seeking support for their own mental health and prefer informal help from colleagues or friends (34, 35). Embedding mental health education within undergraduate curricula has been shown to increase students’ confidence to support peers (36). Given that doctors in all specialties encounter patients with mental health challenges, MHFA may offer a structured, practical approach to developing mental health literacy and preparedness at undergraduate level.

Although originally designed for the general public, MHFA has demonstrated benefits for healthcare students, including nursing (24–26, 30), pharmacy (20, 28–30, 37–39), medicine (18, 21, 30) physiotherapy (22) and paramedic (27, 40). The programme has been shown to improve mental health literacy, confidence, and attitudes while reducing stigma (29, 41).

**Figure 1:**
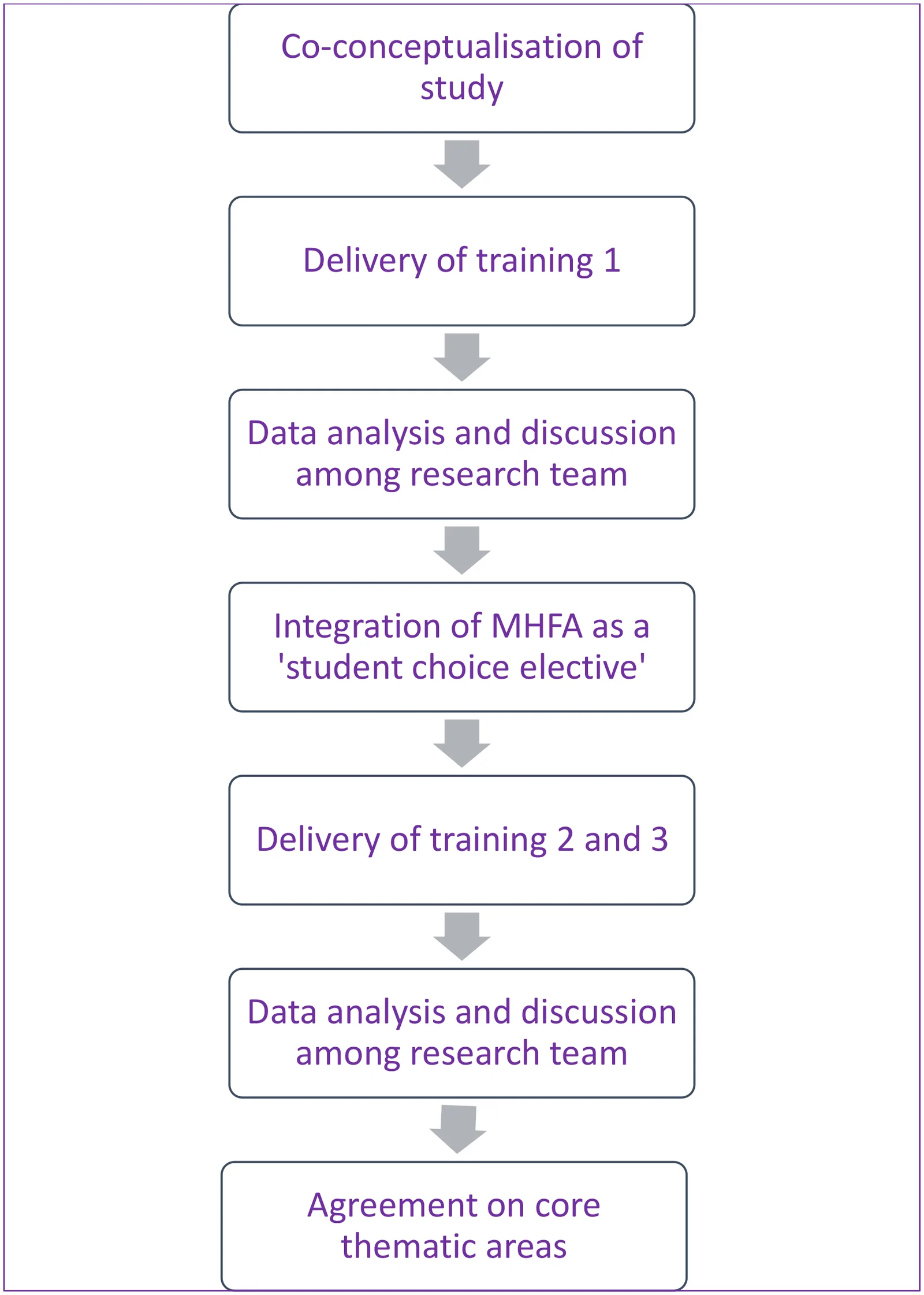
Study design.

In Australia, an online version of MHFA has been developed specifically for health profession students (42). This was launched in 2024 and is developed to prepare students both for their role as future health professionals, promote peer support skills and develop resilience and self-care (**Figure 1**).

It has been found that medical professionals hold more pronounced stigma about mental health than the general public (43–45) and educational initiatives have shown promise to help reduce this (46). This also impacts how likely a medical professional will be to seek help for their own mental health problem. Perhaps contributing to this is the curricular gaps identified in medical education. For example, in the UK less than 2 hours is prioritised for education around eating disorders (47), despite them accounting for the highest mortality rate among all mental illnesses (48). Moreover, similar trends exist for addiction education. An international survey administered by World Health Organisation which included 162 countries found that one-third of all countries reported that they had no medical training in the management of substance use disorders (49). There is also an appetite for recovery focused education in medical education, for example, a study with patients and caregivers’ priorities for mental health education of health profession students highlighted the importance of a recovery focused ethos (50).

### 1.1 Research questions

1. How do medical students experience standard Adult MHFA?
2. How can MHFA be tailored for medical students?
3. What suggestions do medical students have for improving mental health education within medical curricula?

## 2 Materials and methods

### 2.1 Study design

The standards for reporting on qualitative research checklist was used to guide the reporting quality within this manuscript (51). The study emerged from a student–staff partnership initiative within the institution. Two final-year medical students led a pilot MHFA initiative, identifying a perceived gap in mental health skill development within the curriculum. They collaborated with academic staff experienced in MHFA delivery and curriculum integration. Embedding student-led research design aligns with the ‘students as partners’ model, enabling curricula to reflect student priorities and fostering a collaborative educational culture (52, 53).

Traditionally, higher education has favoured a hierarchical structure whereby students learn what teachers wish to teach, and this structure continues to dominate today (53). A recent approach to teaching and learning is the ‘students as partner’s model’. Educators and institutions benefit from this as teaching can be refined based on student perspectives, this can also foster a greater sense of community between students and staff (53).

#### 2.1.1 The research cycle

**Figure 1** demonstrates the collaborative process of the research cycle.

1. Co-conceptualisation of the study: Two final year students proposed the topic of MHFA for medical students as part of the university student engagement programme. On the basis of this proposal, a research team is devised comprising these medical students, a PhD student exploring MHFA for health profession students at the university, and her supervision team who have experience embedding MHFA as a mandatory component of the Masters of Pharmacy at the university. The student partners devise the protocol for the study including the research design, and application for ethical approval is completed.
2. Delivery of training 1: Following ethical approval the first training is delivered as an extra-curricular activity for medical students across years. A focus group is held online with 6 participants a day after the training. The focus group is facilitated by the student partners with the lead author present to support the students.
3. Data analysis and discussion among team: The transcript is imported into NVIVO for thematic analysis. An initial analysis conducted by student partner (KY) and the lead author highlight codes relating to ‘cultural competencies’, ‘mental health literacy’, and ‘opportunity to practice skills’. Coding was inductive only.
4. Integration of MHFA as a student choice elective: Based on the initial findings from the first focus group, the lead and senior authors (ZM and JS) met with the Department of Medicine colleagues responsible for the ‘Student Choice’ elective to discuss the potential of the training. MHFA training is 12 hours long, and the requirement of the electives are 20 hours of teaching over 5 days. Therefore, after discussion between these colleagues and MHFA Ireland, it was decided to break up the MHFA module into 3-hour sessions across 4 days, and hold a ‘simulated practice’ opportunity on the final day.
5. Delivery of training 2 and 3: Trainings 2 and 3 were implemented into this student choice elective for second year medical students. In addition to receiving the MHFA training, these students had the opportunity to practice their skills with each other on the final day. Students participating in the study attended the focus group after the simulated practice.
6. Data analysis and discussion among research team: After these two trainings, all focus group recordings were transcribed by student partners (SA, PJ) who were recruited for this purpose. These transcripts were uploaded into NVIVO and coded as per thematic analysis guidelines (54, 55).
7. Development of thematic areas: The research team met to discuss codes and co-construct the repeating patterns into thematic areas.

### 2.2 Pedagogical framework and learning environment

Irish MHFA training includes an introduction to mental health and early intervention, the ALGEE action framework, depression, anxiety, psychosis, and substance misuse, delivered through didactic, dialogic, and case-based learning. The ALGEE action framework consists of six actions: Approach, Assess and assist, Listen nonjudgementally, Give support and information, Encourage professional help, and Encourage other supports (56).

MHFA was provided at three different time points directly targeting medical students. The 12-hour standard MHFA for adults was delivered by licensed MHFA instructors most of whom are embedded in the university. The first delivery of MHFA was completed over 2-days as an extra-curricular delivery targeting students across years in medicine. This training was delivered by the lead researcher (ZM) and an external MHFA instructor provided by MHFA Ireland. In order to support the goals of the student-researchers, the lead researchers collaborated with The School of Medicine to place MHFA within an elective module. The student choice programme is offered to all first-and-second year medical students. The students are able to select from a wide selection of interesting topics which are designed to enhance medical students’ professional identity and help prepare them for clinical practice. They are delivered over 20 hours and held over 5 days, ending with a presentation or reflective assignment which is graded. The next two MHFA programmes were delivered over 4 days within this student choice’ elective targeting students in their second year of medicine. These two trainings were delivered by embedded MHFA instructors, one of whom is ZM. The standard MHFA programme comprised 12 training hours. For Trainings 2 and 3, a separate 5-hour simulation session and additional elective requirements were included to meet the 20-hour Student Choice elective requirements. No adaptations to the MHFA manual or delivery were made in the delivery of any of the MHFA trainings. **Table 1** outlines the intervention details as per the TIDeiR checklist (57).

**Table 1:** Training implementation of Mental Health First Aid across the study using the TIDeiR checklist.

Item	Training 1	Training 2	Training 3
Brief name of intervention	Standard adult MHFA	Standard adult MHFA	Standard adult MHFA
Why – rationale for intervention	Identified gap in education.	Identified gap in education.	Identified gap in education.
What – materials used in the intervention	MHFA manual provided to all participants. MHFA training is 12 hours and content includes: Introduction to mental health in Ireland, Depression, Anxiety, Psychosis and Substance Misuse.	Same as training 1.	Same as training 1.
Procedures – procedures, activities or processes	Standard MHFA: All sessions involve group activities, case studies, video scenarios and some sessions include lived experienced perspectives through video.	Standard MHFA – Same as training 1. **Additional Simulated practice is included** in this training which is not provided in standard MHFA (5 hours); Simulation included assembling in small groups, reading a scenario and practising being the MHFAider or patient and practicing three different scenarios in this small group setting.	Standard MHFA – Same as training 1. Additional Simulation – Same as training 2.
Who – instructors expertise, background and any specific training given	Delivered by two female, similar age instructors who are licensed MHFA instructors.	Delivered by two embedded MHFA instructors at RCSI. Both female and similar age. One works as a social worker with student welfare team at the university and the other is a former paramedic fire fighter working as a teacher practitioner at the university.	Same as training 2.
How – describe modes of delivery	In person at St John of God University training room in group of 20 participants.	In person at RCSI Dublin campus in group of 20 participants.	In person at RCSI Dublin Campus in group of 20 participants.
Where – type of location where intervention occurred	External location.	On campus.	On campus.
Modifications	The training was delivered over 2 days as per standard adult MHFA.	The training was delivered in 3-hour sessions over 4 days. A simulated practice occurred on day 5. As per the student choice elective requirements, attendance at all 5 days was mandatory, and a 500 word reflective assignment was required to pass the module. This was not graded by anyone involved in this study.	Same as training 2.
How well fidelity to training was maintained	Both instructors are licensed instructors who delivered the training as per MHFA Ireland guidelines.	Both instructors are licensed instructors who delivered the training as per MHFA Ireland guidelines. All students were aware that the day 5 was not part of the standard training,	Same as training 2.

### 2.3 Participant recruitment and data collection

Convenience sampling was used. Students who completed the full 12-hour MHFA programme were eligible to participate. Once ethical approval was received all students registered for the MHFA module received an invitation to participate via the virtual learning environment ‘Moodle’ two weeks before the module began. This invitation directed students to the participant information and consent form. All registered students were invited to post-training focus groups. Three focus groups were conducted with 23 medical students from foundation to final year following the focus group guide developed at the beginning of the study. Focus group 1 was cofacilitated by the lead author, and two student partners. Subsequent focus groups were facilitated by the lead author who was not involved in delivering these trainings.

Focus group facilitators used structured probing techniques to actively invite quieter participants, invited disagreeing or dissenting opinions to the discussion, and set ground rules emphasising that there were “no right or wrong answers.” Co-facilitation by student partners in the initial group helped equalise peer-to-researcher power dynamics and fostered a non-hierarchical space.

In considering sampling adequacy, we drew on the principles of information power (58). While the aim was broad, it was contextualised within a homogeneous population (medical students attending MHFA). No established theory was used to develop the focus group questioning and analysis, however the main aim was indepth analysis of a small group, ensuring that the sampling was sufficient to answer the research questions (58).

#### 2.3.1 Participant safeguarding during training and data collection

A core aspect of MHFA delivery is safeguarding students and creating a space whereby students can advocate for a small break if the content is emotionally challenging. This is discussed at the beginning of the training when devising the ‘group agreement’ which is referred back to throughout the training. In addition to this, the training is delivered by two instructors in tandem. This ensures that if any student does need to leave or take a small break, another instructor is there to support a student if needed. In addition to this, a distress protocol was devised for research purposes which outlined steps the lead researcher should take if a research participant became distressed during a focus group.

### 2.4 Data analysis

Recordings were transcribed verbatim, pseudonymised, and uploaded to NVivo for qualitative analysis. Inductive thematic analysis was conducted following Braun and Clarke (55). Multiple researchers independently generated initial codes before engaging in collaborative discussions regarding interpretation and theme development. Student partners met with the lead researcher before coding to discuss the analytic process and used the Braun and Clarke six steps to thematic analysis to guide the process. The lead researcher also provided the student partners with a worked example of thematic analysis (59) to support their understanding of the methodological approach. **Table 2** below describes the full analytical process using the six steps.

**Table 2:** Thematic analysis steps taken in the study.

Thematic analysis steps	Steps undertaken for the data analysis
Familiarisation with the data	All researchers involved in the analysis phase read and re-read all three transcripts including those that were transcribed by previous student partners.
Generating initial codes	All student partners read the Braun and Clarke paper outlining the thematic analysis process to understand the coding steps. Each researcher involved in this stage independently developed codes in Nvivo. Codes per researcher ranged from 11-35. Researchers discussed code labels and refined terminology to facilitate ongoing analytic conversations across the dataset.
Generating themes	The coded data was reviewed and analysed as to how different codes may be combined into themes. Student partners and the lead researcher discussed and debated the significance of combined codes and their meaning. This process resulted in the generation of four thematic areas with several subthemes identified between researchers; Importance of MHFA - Physician mental health - Understanding patients - Moving beyond the textbooks - Stigma within healthcare settings A multicultural approach to MHFA - Using MHFA in a different culture - Utilising statistics for other countries - Supporting someone’s mental health from a different culture Building psychological safety to learn about mental health - Interactivity - Multimodal teaching and learning - Learning from each other - Open dialogue Motivation to be a MHFAider - Personal experiences
Reviewing potential themes	Transcripts were re-read by student partners and lead researcher with the candidate thematic areas in mind to ensure it matched the data in the transcripts.
Defining and naming themes	This resulted in the thematic areas being refined into three thematic areas with several sub-themes within each. Themes were carefully developed between the researcher and student partners. During theme development, codes relating to instructor-student rapport, opportunities for peer discussion, and interactive group activities appeared conceptually linked. Drawing on prior educational research experience, the lead researcher suggested psychological safety as a potential interpretive framework. The team subsequently revisited the data and determined that this concept meaningfully captured the shared pattern across these codes. The definition and naming of themes were discussed collaboratively with the research team in line with the original research questions and underlying meanings within the data.

### 2.5 Reflexivity

The first author has lived experience of mental health challenges, informed interpretation while enhancing sensitivity to participant narratives. The study was codesigned with final-year medical students who identified gaps in mental health education. Efforts were made to ensure that power dynamics among the team were minimised. The lead researcher, a PhD student met with all student partners to work collaboratively. The lead researcher has not taught or been involved in the grading of student partner’s work. The wider team included expertise in mental health practice and curriculum design, supporting reflexive dialogue throughout. Regular team meeting discussions involved in depth discussion of arising themes and potential alternative explanations. This process led to the refinement of themes to better represent nuanced, ambivalent participant accounts, ensuring that participant accounts remained central to interpretation while researchers critically reflected on the influence of their prior assumptions and experiences. The lead researcher was also a licensed MHFA instructor and was involved in the implementation of MHFA within the medical curriculum. While this provided a detailed understanding of the programme, it also had the potential to privilege positive interpretations of MHFA, which were explored critically during team discussions. This was particularly relevant during development of the psychological safety theme, where the lead researcher’s prior experience in educational research informed consideration of psychological safety as an interpretive lens. This interpretation was subsequently discussed by the research team and re-examined against the dataset.

## 3 Results

Three elective MHFA trainings were delivered to medical students between November 2023 and July 2025. Of the fifty-eight students who chose to undertake MHFA training, twenty-three agreed to participate in the research. Focus groups included 6 to 9 participants with students representing foundation to final year of medicine in the total dataset. Focus group 1 was conducted online with a range of students from foundation to final year, and both focus group 2 and 3 were conducted in person with only second year medical students. Overall, participants were enthusiastic about MHFA training, and considered mental health skills to be a core skill needed in their future profession. All students were full time medical students and of similar age. No demographic information was collected about the participating students and only necessary data was collected. All focus groups were conducted by the lead researcher with a student researcher. Focus group recordings were transcribed by a student researcher. Coding and theme development were undertaken collaboratively by the lead researcher and student researchers through iterative discussion and interpretation of the dataset. At the end of the process, three themes were generated from the data; Mental health literacy is a core competency, *MHFA provides a pedagogy of safety and authenticity*, and optimising MHFA for medical students.

### 3.1 Themes arising from the data

#### 3.1.1 Mental health literacy as a core competency

Across focus groups, participants consistently described mental health literacy as a crucial skill:

“it’s really important for us to first understand the mental status of the patient before trying to fix their physical problem” – Student 2, focus group 2

“I’m in med 2 (second year of medicine) but last year all of us had to do conventional first aid training. So personally, I don’t really see why mental health first aid training shouldn’t be treated the same.” – Student 3, focus group 1

“Mental health is extremely important and this is something we need to take seriously, regardless of what specialty we are going into cardiology, neurology, anything outside of psychiatry, I still feel like you are going to meet a diverse range of patients going though things like that.” – Student 4, focus group 2

Other students identified that there is a gap in their current curricula in relation to developing skills in mental health:

“We don’t go into the patient and the empathetic skills that you would need to have in order to like communicate with them effectively” – Student 3, focus group 3

“I think as a medical student you learn about all these diseases like depression, schizophrenia, if you are able to realise when someone might be suffering with these, but then we don’t really get taught on how to first approach them … what are the correct phrases to use and then like how to take the first steps to talking to them” – Student 5, focus group 2

“This really provides us with a proper framework that I feel like can be applied to a lot of other things and how to properly approach people in a way that they will be more receptive and actually like accept our help” – Student 5, focus group 2

#### 3.1.2 MHFA provides a pedagogy of safety and authenticity

Many students across focus groups suggested that the way the MHFA training is structured was well received:

“It wasn’t just sitting down and listening the whole time, we were actually able to be more interactive, I really appreciated that” – Student 4, focus group 2

“I really liked the amount of mixed materials, the video clips and portrayals (of people with mental illness), those things really stick in your mind” -Student 5, focus group 3 “Because we are in healthcare, we have to apply a lot of things that we learn, the best part of the course, and I am not sure if it was just the instructors but after every video, they would make us apply ALGEE to the real life, which I thought ingrained it better into our heads over the two days” – Student 2, focus group 2

Other students really valued how MHFA training allows for collaborative discussions and open dialogue about mental health:

“It was great to get everyone’s point of view on different things so that you get different perspectives” – Student 4, focus group 2

“I did not know anyone (here) before, so I feel like the (discussions) brought us closer together and we learned so much about each other, and that was such a nice aspect of it” – Student 5, focus group 3

Many students across focus groups opened up about their own experiences with mental illness, or supporting someone they knew through mental illness:

“I am grateful that I know ALGEE now so I have a second chance to not hesitate in approaching people” – Student 7, focus group 2

“There are a lot of people with different mental illnesses in my family, and that was difficult to deal with because we didn’t have the knowledge of what it was”…“So it has been wonderful to be here learning these effective skills and it feels like I have claimed something back” – Student 5, focus group 2

Students also commented how the structure of MHFA coupled with the rapport built from the instructors made the learning space safe:

“They (the instructors) made it a really good safe space to learn and gather perspectives” – Student 1, focus group 1

“They (the instructors) made us comfortable to open up to each other too” – Student 5, focus group 2

“I liked the ice breaker at the beginning with throwing the ball to each other so that we could introduce each other, it made the training a lot more personal” – Student 7, focus group 3

“The instructors were really interactive, were really respectful of all of our opinions, and boundaries” – Student 3, focus group 2

#### 3.1.3 Optimising MHFA for medical students

Several students across focus groups mentioned how eating disorders was missing from the taught content:

“It would be really useful if eating disorders was to be added, because especially for the youth it’s something that is actually a huge problem, there’s a lot of like fat shaming, you know there’s just this like ideal body type that you’re supposed to have. It’s something that impacts a lot of people worldwide” – Student 1, focus group 1

“I think eating disorders are very important to discuss and I think they would be good to be on the course” – Student 6, focus group 3

Other students specifically mentioned personal experiences with eating disorders:

“I’ve always been interested in mental health, but at my high school, I went to all girls high school, so, it was just like crazy eating disorders everywhere” – Student 8, focus group 2

“My sister struggled with an eating disorder. So that was definitely also another factor. And it’s also just always been really interesting to me as to how like the family and other people always react, and I feel like that kind of like helped with my like interest in mental health” – Student 6, focus group 3

Additionally, students recommended that the safety of the MHFAider could be included in an updated version:

“More emphasis on your own safety when you’re doing mental health first aid” – Student 2, focus group 3

Other students recommended that given the particular risk factors of physician burnout, that it is essential that medical students learn practical skills for self-care and building their own capacity for wellbeing:

“Just recently so many statistics have come out about residency and mental health risks, I think it’s so crucial to have a bit of an awareness of that and like how you are like managing your own stress” – Student 1, focus group 3

“We’re going to be future healthcare professionals but that doesn’t exclude us from the conditions as well, there is a chance that our colleagues could have a mental health breakdown” – Student 3, focus group 1

## 4 Discussion

In this study we explored medical students’ perceptions of MHFA. The results showed that medical students feel that developing mental health literacy is an essential skill, and that medical students are highly receptive to MHFA as a potential initiative for developing this literacy.

The study results show that medical students own perceptions of the mental health literacy (MHL) they develop within the medicine curricula needs attention. The concept of MHL has been defined by Jorm as the knowledge as belief about mental health challenges which aid their recognition, management or prevention (60). Mental health literacy also encompasses knowing about help-seeking options and treatments available for people in mental distress (60). The students who proposed this study were final year students who considered the current medical curricula to be absent of developing this crucial skill. This matches up with recent studies on the mental health literacy of health profession students which has found a significant deficit in this area (61). Studies consistently show that medical students MHL scores are low, comparable to that of general public (62–64). Whilst this study did not utilise any instruments to measure the student’s mental health literacy before or after the training, many previous studies have reported improvements on participants MHL after taking MHFA training (15, 22, 31, 65, 66).

One of the themes arising in the data was the pedagogical design of MHFA. All content is designed in partnership with experts by experience through a Delphi consensus design. Video narratives of lived experiences are also spread throughout the training enabling participants to hear accounts of real experiences of recovery.

Another factor influencing participants’ engagement with MHFA appeared to be the student characteristic of previous lived experience with mental illness (67). Participants described that this lived experience was the reason that many students wanted to learn MHFA. It has been found that those who have personal experiences of mental illness or who have a close friend or family member with mental illness, tend to have greater mental health literacy (62). Many of the students across focus groups suggested that learning about physician mental health and increasing the focus on self-care strategies and support was a priority for them. It was understood among students after the training that they too might experience some of the mental health challenges discussed during the training and it was imperative that they understand how to support each other. This has been previously highlighted in other research which cited that health professionals need to develop skills in supporting their own well-being (50). Whilst MHFA does not leave room for in depth education on personal wellbeing and self-care, this might be suitably addressed in other professional and personal development education. This concern has also previously been highlighted by WHO (1) and medical professionals (10) who call for mental health education initiatives to be implemented at undergraduate level.

Another aspect of the pedagogy of MHFA is the development of a psychologically safe learning environment. This theme was inductively derived from the data. Psychological safety (PS) is a concept long recognised within quality patient safety and improvement (68, 69), however has more recently been addressed within medical education. PS exists when individuals feel free to take risks, raise problems, disagree, ask questions, and admit mistakes (68). A paper bringing together key constructs of PS in education found that having a supporting relationship with peers and mentors was crucial to developing that PS within educational settings (70). According to our participants, the facilitators building opportunities for students to get to know each other and their motivations for selecting MHFA was crucial for developing this PS environment.

The presence of PS within MHFA for medical students is notable, as previous studies have found PS to be absent within many learning environments for medical students (71) and there is an increasing understanding that PS can support student learning and engagement (72). According to McClintock (73) there are three tasks that an educator can include to promote a PS learning environment. These are: setting the stage, inviting participation, and responding productively (73). Within the MHFA training, it is an important part of the introduction that everyone introduces themselves, a group agreement is devised and expectations between learners and educators are expressed. Additionally, a core aspect of MHFA is the participation of students with each other in group activities, and small and whole group discussions. MHFA is also all about understanding that stigma and misunderstanding exists and encouraging open discussion among students during the training.

### 4.1 Limitations

While this study provides valuable qualitative insights into the integration of MHFA training for medical students, several limitations must be acknowledged. First, this was an exploratory qualitative study conducted within a single-institution setting, which may limit the generalisability or transferability of the findings to other medical schools with different cultural or curricular structures.

Second, the study did not include pre- or post-intervention quantitative measures of mental health literacy or stigma, preventing any objective evaluation of the training’s measurable efficacy. Furthermore, the lack of longitudinal follow-up means it is impossible to determine whether the students’ perceived benefits, self-care strategies, and peer-support skills were sustained as they transitioned into intense clinical environments.

Third, our participant recruitment introduces several interconnected biases. Recruitment was entirely voluntary, creating a distinct self-selection bias. It is highly probable that students who chose to enrol in the training and subsequent focus groups possessed a pre-existing interest in mental health, or had personal experiences with mental illness, making them more likely to participate than the wider student body.

While focus groups stimulated rich collaborative discussion among medical students, the group format introduces inherent methodological limitations. Despite active facilitation to balance airtime and manage dominant voices, peer influence, social desirability, and implicit medical hierarchy (e.g., junior foundation students alongside final-year peers) may have encouraged conformity or muted non-mainstream viewpoints. Moreover, some participants may have withheld highly sensitive personal experiences or critical feedback regarding institutional mental health provision that they might otherwise have disclosed in individual, confidential depth interviews Finally, the qualitative data collection may have been influenced by social desirability bias, as students might have answered in ways they felt were expected in a medical education context.

## 5 Conclusion

This study explored medical students’ perceptions of Mental Health First Aid (MHFA) training and their views on how mental health education could be strengthened within undergraduate medical curricula. Participants viewed MHFA positively and described it as a valuable opportunity to develop mental health literacy, discuss mental health openly with peers, and reflect on supporting both patients and colleagues experiencing mental health challenges.

Three themes were developed from the data: mental health literacy as a core competency, MHFA as a pedagogy of safety and authenticity, and opportunities to optimise MHFA for medical students. Participants highlighted the value of interactive learning approaches and identified areas where additional content may better reflect the needs of medical students.

These findings suggest that MHFA may represent a useful educational approach within undergraduate medical education. Further research is required to evaluate its impact on mental health literacy, stigma, help-seeking behaviours, and longer-term educational and professional outcomes.

## Data Availability

The raw data supporting the conclusions of this article will be made available by the authors, without undue reservation.
